# Shear Bond Strength of Simulated Single-Retainer Resin-Bonded Bridges Made of Four CAD/CAM Materials for Maxillary Lateral Incisor Agenesis Rehabilitation

**DOI:** 10.1055/s-0043-1776335

**Published:** 2023-12-29

**Authors:** Maria João Calheiros-Lobo, João Mário Calheiros-Lobo, Ricardo Carbas, Lucas F. M. da Silva, Teresa Pinho

**Affiliations:** 1 UNIPRO - Oral Pathology and Rehabilitation Research Unit, University Institute of Health Sciences (IUCS-CESPU), Gandra, Portugal; 2 Conservative Dentistry, Department of Dental Sciences, University Institute of Health Sciences (IUCS(IUCS-CESPU), Gandra, Portugal; 3 Dental Prosthetist, Private Prosthesis Laboratory, São Mamede Infesta, Portugal; 4 Department of Mechanical Engineering, Faculty of Engineering, University of Porto, 4200-465 Porto, Portugal; 5 INEGI - Institute of Science and Innovation in Mechanical and Industrial Engineering, University of Porto, Porto, Portugal; 6 Institute for Molecular and Cell Biology (IBMC), Institute of Innovation and Investigation in Health (i3S), University of Porto, 4200-135 Porto, Portugal

**Keywords:** 3D additive manufacturing, adhesion, CAD/CAM, maxillary lateral incisor agenesis, monolithic ceramics, shear bond strength, surface energy

## Abstract

**Objectives** Maxillary lateral incisor agenesis (MLIA), treated orthodontically by space opening, requires complimentary aesthetic rehabilitation. Resin-bonded bridges (RBBs) can be equated as interim rehabilitation until skeletal maturity is achieved to place an implant-supported crown or as definitive rehabilitation in case of financial restrictions or implant contraindications. Scientific evidence of the best material must be confirmed in specific clinical situations. Computer-aided design and computer-aided manufacturing (CAD/CAM) materials are promising versatile restorative options. This study aimed to identify a straightforward material to deliver interim or definitive RBBs for nonprepared tooth replacement in MLIA.

**Materials and Methods** Single-retainer RBB made from CAD/CAM ceramic blocks (Vita Enamic [ENA], Suprinity [SUP], and zirconia [Y-ZPT]) and a three-dimensional (3D) printed material (acrylonitrile butadiene styrene [ABS]) were evaluated by shear bond strength (SBS) and mode of failure, after adherence to an artificial tooth with RelyX Ultimate used in a three-step adhesive strategy.

**Statistical Analysis** The load to fracture (N) was recorded, and the mean shear stress (MPa) was calculated with standard deviations (SD) for each group and compared between materials using boxplot graphics. One-way analysis of variance (ANOVA) followed by the Tukey–Kramer post hoc test was used to compare the differences ( *α* = 0.05). A meta-analysis focusing on CAD/CAM materials evaluated the magnitude of the difference between groups based on differences in means and effect sizes ( *α* = 0.05; 95% confidence interval [CI]; *Z* -value = 1.96). Failure mode was determined by microscopic observation and correlated with the maximum load to fracture of the specimen.

**Results** The mean ± SD SBS values were ENA (24.24 ± 9.05 MPa) < ABS (24.01 ± 1.94 MPa) < SUP (29.17 ± 4.78 MPa) < Y-ZPT (37.43 ± 12.20 MPa). The failure modes were mainly adhesive for Y-ZPT, cohesive for SUP and ENA, and cohesive with plastic deformation for ABS.

**Conclusion** Vita Enamic, Suprinity, Y-ZPT zirconia, and 3D-printed ABS RBBs are optional materials for rehabilitating MLIA. The option for each material is conditioned to estimate the time of use and necessity of removal for orthodontic or surgical techniques.

## Introduction

Maxillary lateral incisor agenesis (MLIA) is a prevalent nonsyndromic congenital tooth agenesis, often bilateral, 1 2 and associated with reduced maxillary sagittal growth and altered relative lower incisor position, 3 making functional postorthodontic stabilization pertinent. Its challenging treatment has valuable aesthetic options, including orthodontic space opening followed by lateral incisor prosthetic replacement or space closure with canine mesialization complemented by tooth remodeling. 4 5 Single-retainer resin-bonded bridges (RBBs) are aesthetic, minimally invasive restorative, and reversible options for interim or definitive rehabilitation in cases of space-opening procedures, 4 5 mainly in clinical situations with ongoing maxillofacial growth due to periodontal and aesthetic factors. 6 7 8

Computer-aided design and computer-aided manufacturing (CAD/CAM) materials are versatile for aesthetic restorations, but clinical evidence-based data concerning their success and durability still need to be explored. 9 10 Available for digital workflow, these industrial materials evolve faster than the data returned from high-quality clinical trials, 11 leading clinicians and dental prosthetics to doubts about optimizing the available options. 12 Furthermore, in vitro studies usually integrate equipment unavailable in clinical settings, and only some experimental protocols can be transposed directly from the laboratory to the clinical context, 11 making pertinent experimental designs that simulate clinical situations and settings and uses standardized base adherends to avoid the heterogeneity of the natural teeth.

CAD/CAM monolithic ceramics are mainly polycrystalline, glass-matrix, indirect composites, and hybrid ceramics. 13 14 The polycrystalline ceramic Vita YZ HT (Y-ZPT) (VITA Zahnfabrik, Bad Säckingen, Germany) is a 5 mol% yttria-stabilized zirconia and a standard for new generations by its physical and mechanical characteristics (opaque white appearance and high flexural strength/1,200–1,500 MPa). 15 16

Combining a low flexural modulus with a high flexural strength (150–160 MPa), the hybrid ceramic Vita Enamic (ENA; VITA Zahnfabrik) is a polymer-infiltrated ceramic network 13 capable of elastic deformation before failure, with a mechanical behavior similar to that of a human tooth. 17 Despite the low stiffness, 18 it is quite stable under extreme acid exposure, and cyclic loading does not affect its properties. 19 Its unique polymer-based microstructure is essential for the micromechanical bond and the performance of the adhesive interface 20 21 due to a decreased crack propagation. 22 High translucency, fluorescence, and opalescence are the main characteristics of Vita Suprinity (SUP; VITA Zahnfabrik), according to the manufacturer. This homogeneous fine-grained glass-ceramic enriched with zirconia has a consistently high load capacity (flexural strength in crystallized state, 420 MPa). Delivered precrystallized, it is an interesting material for anterior RBBs because of its aesthetics, biocompatibility, mechanical properties, and more straightforward adhesive protocol. 13 14 23 Clinical data remain scarce, often controversial, and limited to short-term observational periods. 24 25

Medical ABS (acrylonitrile butadiene styrene; Smart Materials 3D, Jaén, Spain; ISO 10993–1) is a lightweight bisphenol A (BPA) free thermoplastic polymer that attains tensile strengths ranging from 15 to 38 MPa with an elastic modulus of 1,300 to 1,800 MPa. Produced via fused deposition, its mechanical behavior depends on processing temperatures, printing parameters, proportions of monomers in the ABS structure, and force orientation during testing. 26

The quality of an adhesive joint is determined by the bond quality at different interfaces and the adhesive strength of the restorative materials, as in the case of RBBs. The interfaces between the dental tissue and the adhesive cement and the connection between the cement and the surface of the restorative material play essential roles. 27 In this process, adhesion and cohesion 23 28 are involved, with the first between the substrates and the second within each substrate.

Characterization of the interface before adhesion, during function, and after failure is helpful in adhesive joint research and remains a significant challenge 28 because a specific adhesive protocol is required for each paired material to obtain the highest bond strength. 11 29

Advances in adhesive dentistry and technology expanded the use of RBBs with alternative preparation designs and materials. 30 To best predict the future clinical performance of CAD/CAM materials to fabricate RBBs, similar designs and fabrication procedures following real dental laboratory and clinical procedures should be chosen. 31 Meanwhile, it is accepted that the adhesive strength of zirconia depends on particle abrasion and primers or adhesives containing 10-methacryloxydecyl dihydrogen phosphate (MDP). 32 33

This study evaluated single-retainer RBBs manufactured similarly to those for clinical application in MLIA. Three CAD/CAM monolithic ceramics and one additive-manufactured CAD/CAM material adhered to an artificial tooth with dual-cured cement were assessed for shear bond strength (SBS) and fracture mode. The null hypothesis was that no differences would be observed between the SBS of the tested materials in the tested RBB model.

## Materials and Methods

The materials used in this study are listed in Table 1 . Polycrystalline zirconia (Y-ZPT) was used as control material. Based on previous research, 21 a photoinitiated dual-cured adhesive cement, RelyX Ultimate (RU; 3M ESPE, Seefeld, Germany), used in a three-step adhesive strategy, was used to adhere the experimental RBBs to an artificial tooth.

**Table 1:** General description of materials used in this study, their compositions, and manufacturers

Material	Name	Code	Composition	Manufacturer
CAD-CAM monolithic ceramics	Vita Enamic	ENA	86% feldspar ceramic: SiO _2_ 58–63%, Al _2_ O _3_ 20–23%, Na _2_ O _9_ –11%, K _2_ O _4_ –6% by weight, 14% polymer by weight: TEGDMA, UDMA	VITA Zahnfabrik, Bad Säckingen, Germany
	Vita Suprinity	SUP	Zirconium oxide 8–12%, silicon dioxide 56–64%, lithium oxide 15–21%, various > 10% by weight	VITA Zahnfabrik
	Vita 5Y-TPZ Color	Y-ZPT	Zirconia reinforced with 5% yttria	VITA Zahnfabrik
CAD-CAM 3D-printed material	Medical ABS	ABS	Acrylonitrile butadiene styrene	Smart Materials 3D, Jaén, Spain
Resin-matrix composite cement	RelyX Ultimate	RU	MDP phosphate monomer, dimethacrylate resins, HEMA, Vitrebond copolymer filler, ethanol, water, initiators, silane	3M Oral Care, St. Paul, MN, United States
Etching agent	Porcelain Etch Gel	PEG	Hydrofluoric acid 9.6%	Pulpdent, Watertown, MA, United States
Ceramic primer	Monobond Plus	MB	50–100% ethanol, disulfide methacrylate, ≤2.5% phosphoric acid dimethacrylate, ≤2.5% 3-trimethoxysilylpropyl methacrylate	Ivoclar Vivadent AG, Schaan, Liechtenstein
Adhesive system	Scotchbond Universal adhesive	SB-U	MDP, Bis-GMA, phosphate monomer, dimethacrylate resins, HEMA, methacrylate-modified polyalkenoic acid copolymer, filler, ethanol, water, initiators, silane-treated silica	3M Oral Care
Hydrophobic resin	Heliobond	HEL	HEMA, Bis-GMA, UDMA, initiators (camphorquinone and benzoyl peroxide), fillers (silica, glass particles), and solvents (ethanol and acetone)	Ivoclar Vivadent AG
Artificial teeth	Frasaco tooth	FRA	Melamine-based composition	Frasaco GmbH, Tettnang, Germany

Abbreviations: HEMA, hydroxyethylmethacrylate, MDP, 10-methacryloyloxydecyl dihydrogen phosphate; TEGDMA, UDMA, urethane dimethacrylate; triethylene glycol dimethacrylate.

### Acquisition and Processing of Digital Images

**Fig. 1:**
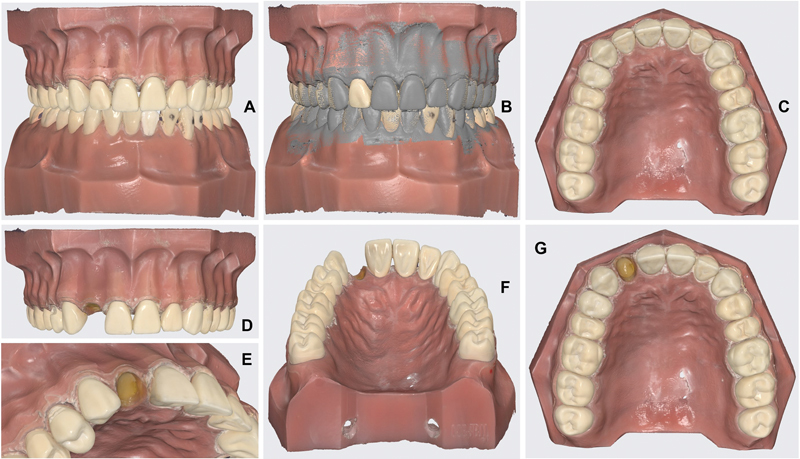
Images acquired using an intraoral scanner. ( **A** ) Reference data from both maxillaries in frontal view, ( **B** ) occlusion data, ( **C** ) reference maxilla in occlusal view, ( **D** ) maxilla simulating a lateral incisor agenesis, ( **E** ) the same in detail, ( **F** ) a view from palatal, and ( **G** ) maxilla simulating a lateral incisor agenesis in occlusal view.

Digital images of a Frasaco A3 Adult Typodont (Frasaco GmbH, Tettnang, Germany) were acquired using a Medit i700 intraoral scanner (MEDIT Corp., Seoul, Republic of Korea) and processed using the software Medit Link v3.0.6 Build 286, and Medit Scan for Clinics v1.9.6 Revision 268 (MEDIT Corp.; Fig. 1 ).

**Fig. 2:**
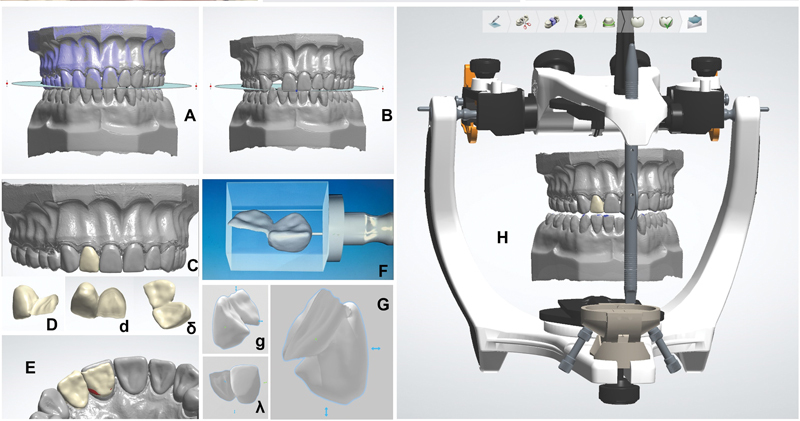
Stereolithography (STL) images uploaded to 3Shape software. ( **A,B** ) Reference data from both maxillaries in frontal view (with and without the lateral incisor) for the calibration of the occlusal plane. ( **C,D,d,δ** ) Details of the planned resin-bonded bridge (RBB). ( **F,G,g,λ** ) Details of the planned RBB to be milled from an ENA block. ( **H** ) The digital case mounted in the digital articulator.

Fig. 2 shows the main steps of the data processing of the digital workflow (3Shape CAD/CAM software, Copenhagen, Denmark), focusing on material resistance and occlusal contacts. The connector area was set at 6.6 mm ^2^ , limited by the vestibular, incisal, and gingival parameters. The minimum thickness for the retainer wing was 0.5 mm. This procedure was repeated according to the manufacturer's instructions for each monolithic CAD/CAM ceramic.

### Single-Retainer Bridge Production

After the digital design, monolithic RBBs were fabricated using a CAD-CAM inLab milling machine (Dentsply/Sirona, Charlotte, NC, United States), following the manufacturer's laboratory procedures. By fused deposition, Medical ABS RBBs were constructed using a Pro2 3D printer (Raise3D, Irvine, CA, United States).

### Cementation of the Resin-Bonded Bridges

Frasaco right central incisors (Frasaco GmbH) were used as adherends. As in a clinical context, the superficial glossy surface was removed using a coarse diamond bur simulating the intraoral removal of the aprismatic or fluoridated enamel, followed by surface conditioning for 60 seconds with 5% hydrofluoric acid. The prepared teeth were shuffled to ensure randomization and operator blinding. A 20-second oil-free air/water spray removed the produced debris. Table 2 lists the adhesive protocols used for each type of material. RelyX Ultimate cement was applied using a three-step adhesive strategy and allowed to self-cure for 7 minutes after 5 seconds of photoinitiation (Elipar S10 curing unit, 1,200 mW/cm ^2^ ; 3M ESPE) through the buccal and palatal sides of the Frasaco tooth. All the steps were performed by the same restorative dentist (single operator) with greater than 30 years of clinical experience.

**Table 2:** Materials used for adherends' surface treatment and adhesion

Cement	Substrate	Surface treatment (Frasaco tooth)	Surface treatment (RBB)	Adhesive system
RelyX Ultimate	ABS	5% hydrofluoric acid	Heliobond	Scotchbond Universal
	Enamic	5% hydrofluoric acid	9.6% hydrofluoric acid 60 s	
	Suprinity	5% hydrofluoric acid	9.6% hydrofluoric acid 20 s	
	Y-ZPT	5% hydrofluoric acid	Al _2_ O _3_ sandblasting	

Abbreviations: ABS, acrylonitrile butadiene styrene; RBB, resin-bonded bridge.

### Mechanical Testing of Resin-Bonded Bridges

**Fig. 3:**
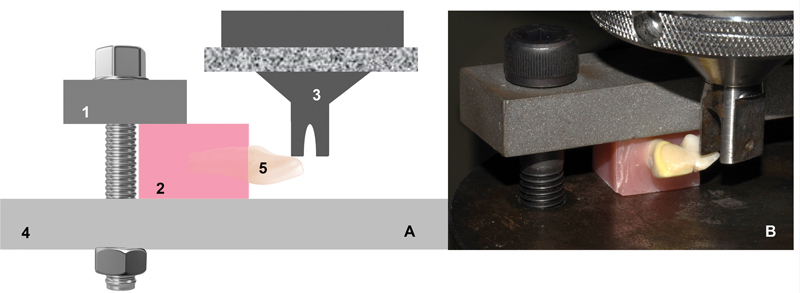
( **A** ) Scheme of the components designed for testing (1, block stabilizer; 2, base adherend incorporated in acrylic resin block; 3, load cell and piston; 4, stationary base; 5, resin-bonded bridge [RBB] to be tested). ( **B** ) Photograph of the shear bonding test with block stabilized on the stationary base and RBB tooth positioned for shear bond strength (SBS) with the piston positioned 2 mm away from the incisal border.

The four groups of RBB specimens (Y-ZPT, SUP, ENA, and ABS) were mechanically assessed under load displacement of 0.2 mm/min (Instron–Universal tensile machine). Fig. 3A and B shows details of the shear-bond test settings. Load-displacement curves were recorded during the mechanical test. The maximum load in the test was used to identify the experimental RBB that supported the highest shear stress, and the highest shear stress supported by the adherend before cohesive failure.

### Data Analysis

The load to fracture (N) and mean shear stress (MPa) with standard deviations (SD) registered for each group were compared using boxplot graphics. One-way analysis of variance (ANOVA) followed by the Tukey–Kramer post hoc test was used to compare the differences ( *α* = 0.05). A meta-analysis focusing on CAD/CAM materials evaluated the magnitude of the difference between groups based on differences in means and effect sizes ( *α* = 0.05; 95% confidence interval [CI]; *Z* -value = 1.96) using a software program (Stata v18.0; StataCorp, College Station, TX, United States). The failure mode was determined by microscopic observation and correlated with the maximum load to fracture of the specimen.

## Results

**Fig. 4:**
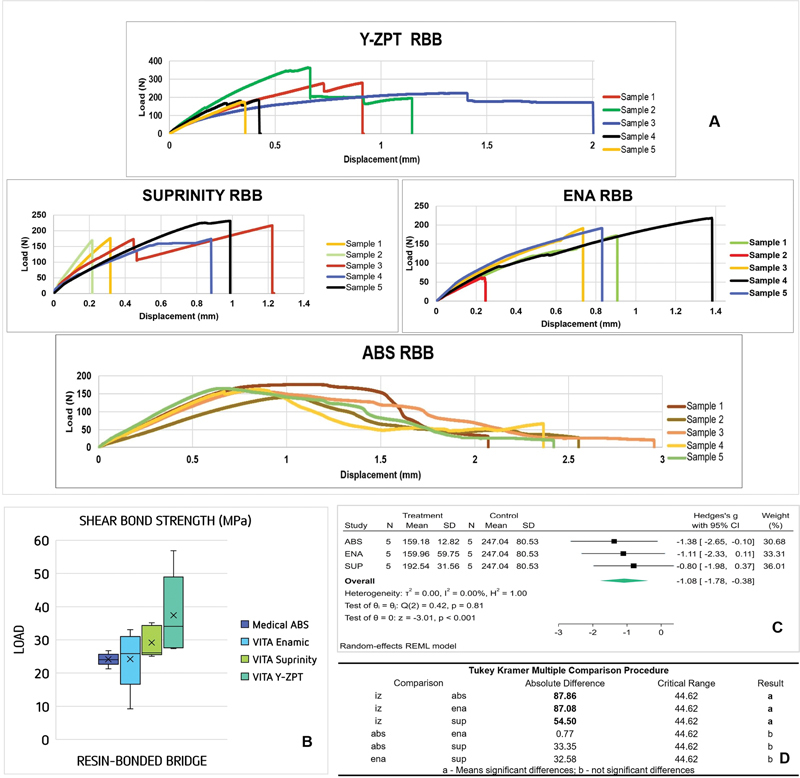
( **A** ) Specimens behavior under load, from the control group (Y-ZPT), Suprinity, Enamic, and ABS groups. ( **B** ) Box plots of shear strength (MPa) of resin-bonded bridges (RBBs) by type of material. ( **C** ) Forest plot summarizing the effect size of the Computer-aided design and computer-aided manufacturing (CAD/CAM) materials and ( **D** ) comparative procedure between groups after analysis of variance (ANOVA).

The mechanical behavior, SBS, and failure mode results are shown in Fig. 4A and Table 3 . Despite having a lower performance, the ABS was more consistent, and observing the curve during loading suggested a marked plastic deformation before failure. Box plots in Fig. 4B allows rapid visualization of the different mechanical performance between materials. The compared mean ± standard deviation values for the adhesive strength were ENA (24.24 ± 9.05 MPa) < ABS (24.01 ± 1.94 MPa) < SUP (29.17 ± 4.78 MPa) < Y-ZPT (37.43 ± 12.20 MPa).

**Table 3:** Compression strength and mode of failure by group and sample

Groups		Compression strength			Mode of failure			
		N	MPa	Sample	AD	C_A	C_RBB	
RelyX Ultimate	Medical ABS	158.45	24.01	1	x		x	
	Medical ABS	176.22	26.70	2	x		x	
	Medical ABS	140.36	21.27	3	x		x	
	Medical ABS	158.28	23.98	4	x		x	
	Medical ABS	162.60	24.64	5	x		x	
	**Failure load**		**Shear strength**					
	**Mean (N)**	**SD (N)**	**Mean (MPa)**	**SD (MPa)**				
	159.18	12.82	24.12	1.94				
					**AD**	**C_A**	**C_RBB**	
	Vita Enamic	170.52	25.84	1	x		x	
	Vita Enamic	61.09	9.26	2	x		x	
	Vita Enamic	158.45	24.01	3		x	x	
	Vita Enamic	191.42	29.00	4			x	
	Vita Enamic	218.30	33.08	5		x	x	
	**Failure load**		**Shear Strength**					
	**Mean (N)**	**SD (N)**	**Mean (MPa)**	**SD (MPa)**				
	159.96	59.75	24.24	9.05				
					**AD**	**C_A**	**C_RBB**	
	Vita Suprinity	171.75	26.02	1	x		x	
	Vita Suprinity	172.16	26.08	2		x	x	
	Vita Suprinity	221.43	33.55	3		x	x	
	Vita Suprinity	165.31	25.05	4	x		x	
	Vita Suprinity	232.03	35.15	5		x	x	
	**Failure load**		**Shear strength**					
	**Mean (N)**	**SD (N)**	**Mean (MPa)**	**SD (MPa)**				
	192.54	31.56	29.17	4.78				
					**AD**	**C_A**	**C_RBB**	
	Vita Y-ZPT	271.40	41.12	1	x	x		
	Vita Y-ZPT	375.01	56.82	2	x	x		
	Vita Y-ZPT	224.90	34.08	3	FTF	FTF		
	Vita Y-ZPT	180.4	27.33	4	x			
	Vita Y-ZPT	183.49	27.80	5	x			
	**Failure load**		**Shear Strength**					
	**Mean (N)**	**SD (N)**	**Mean (MPa)**	**SD (MPa)**				
	247.04	80.53	37.43	12.20				

Abbreviations: AD, adhesive failure; C_A, adherend cohesive failure; C_RBB, bridge cohesive failure; FTF, Frasaco tooth fracture; SD, standard deviation.

**Fig. 5:**
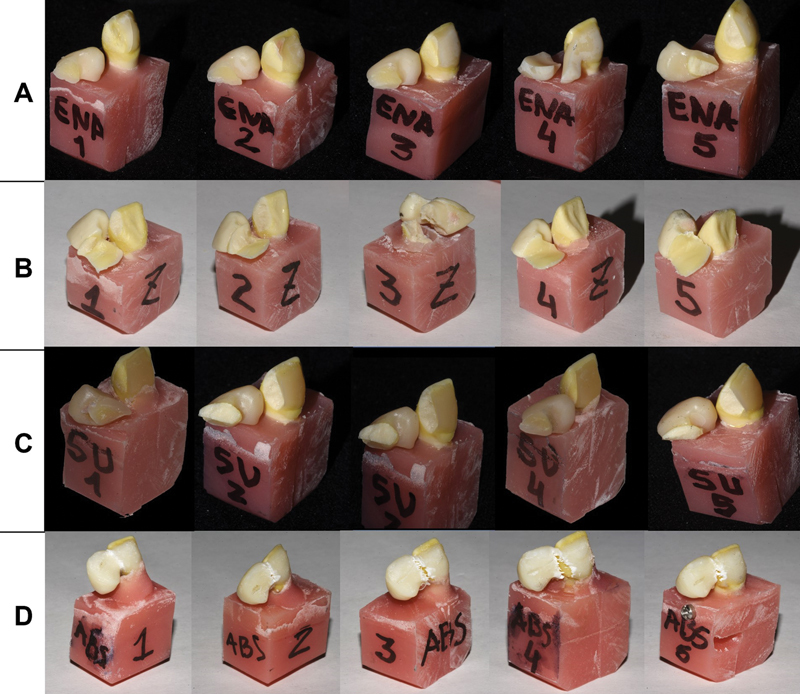
Resin-bonded bridges (RBBs) after testing. ( **A** ) Enamic, ( **B** ) Y-ZPT, ( **C** ) Suprinity, and ( **D** ) ABS groups, with different mechanical behavior after shear load.

**Fig. 6:**
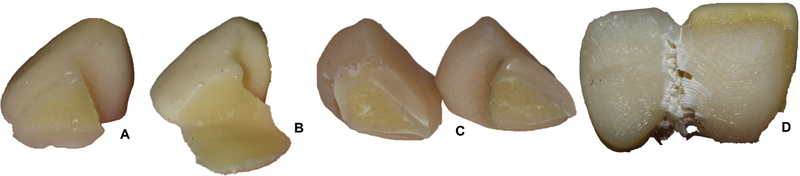
Details of fractured resin-bonded bridges (RBBs) and more frequent failure modes by material type. ( **A** ) ENA, adhesive interproximal and cohesive in retainer. ( **B** ) Y-ZPT, adhesive, with RBB integrity. ( **C** ) SUP, cohesive in Frasaco tooth and retainer. ( **D** ) ABS, adhesive in interproximal, cohesive with plastic deformation in RBB (no RBB tooth loss occurred).

Fig. 4C shows that the mechanical performance of Y-ZPT was significantly better than that of the others ( *p* < 0.001). Fig. 4D shows the results of the compared differences ( *α* = 0.05), highlighting the superior shear strength of Y-ZPT, particularly with ENA and ABS. The failure modes were mainly adhesive for Y-ZPT, cohesive in the RBB for SUP and ENA, and cohesive with plastic deformation of the RBB for ABS ( Figs. 5 and 6 , and Table 3 ).

## Discussion

The null hypothesis that no differences would be found in the SBS among the tested materials in the tested RBB model was rejected because significant differences existed ( *p* < 0.01). Y-ZPT (control) was the most rigid material in this experimental model, consistent with the literature. The ABS, ENA, and SUP groups exhibited consistent mechanical performances.

When speaking about the longevity of rehabilitative treatment, one implicitly thinks of definitive rehabilitation. However, when treating a case of MLIA, rehabilitation must often be temporary and adaptable. This is the case of orthodontic space opening, in which success is reflected in the progressive diastema between the central incisor and the canine tooth. In these specific cases, zirconia RBB is not advisable because it is too resistant to be removed repeatedly without damaging the supporting tooth and has a laborious adhesive technique that hinders the addition of resin-matrix-based materials. Thus, the possibility of fabricating RBBs with materials that are easier to handle, can be replaced at low cost, or are easier to remove from the supporting tooth led us to look for alternatives, mainly focusing on managing orthodontic treatments using aligners that would benefit from a straightforward handling prosthesis.

Adhesion between CAD/CAM materials and teeth substrates depends on adhesive systems 34 and chemical interactions that occur between functional monomers and tooth components, 35 which in turn depend on the properties of the materials, which are crucial to the success of adhesive restorations. 22 In this experimental setting, RelyX Ultimate cement was used based on recent research 21 23 by its adhesive efficiency and versatility when paired with Y-ZPT, ENA, and SUP ceramics. Also, adhesive protocols followed literature guidelines. 11 13 27 33 34 Nothing was found in the literature about the adhesive protocol for Medical ABS. Considering the chemical composition and ease of handling of the material, an old and well-known hydrophobic resin (Heliobond, Ivoclar Vivadent AG, Schaan, Liechtenstein) was selected to simplify the adhesive protocol bearing in mind the possibility of using Medical ABS RBBs as an easily exchangeable interim prosthesis. These adhesive systems evidenced differences between materials' mechanical behavior and allowed them to attain the maximal cohesive strength of the adherend. Therefore, should not have biased the study results.

Regarding CAD/CAM materials, the results showed that the mechanical behavior of RBBs depends on the type of material. Besides that, anterior RBBs have their clinical survival dependent on the mechanical resistance of the connector, which is related to its thickness and bonding surface area. These two variables are especially critical in RBBs replacing lateral incisors due to occlusogingival and vestibular-palatal anatomic restrictions related to the available interocclusal space for the restorative material. About this subject, the literature found is mainly focused on zirconia-veneered frameworks and the posterior region, 36 and a single retainer with a connector diameter of 16 mm ^2^ in the anterior region and 20 mm ^2^ in the posterior region has been suggested with the assumption that periodontal hygiene might be a concern. 37 In the present study, the connector area was set at 6.6 mm ^2^ and the bonding surface area at 42 mm ^2^ following more realistic dimensions suggested in the literature 38 (>4.5 and > 35 mm2, respectively) estimated for the Y-ZPT RBB (control) and consequently for the other materials tested. A recent study 39 using finite elements proposes a volume of 9.04 mm ^3^ instead of an area for an anterior connector planned for lithium disilicate, a material less resistant than zirconia. In any case, there is evidence that an increased cross-sectional thickness of the connector is desirable. 40 Replacing a lateral incisor originates a relatively short cantilever length, which favors the clinical survival of the restoration. 41

Using Frasaco teeth as adherends was very useful because they have a standardized composition and anatomy, allowing the elimination of bias originating from biological factors or different macroanatomies of the palatal face of a natural incisor, which can occur if natural teeth have been used, as only slight asperization was intended, as in a minimally invasive approach. 8 A practical comparison between the materials used to manufacture single-retainer RBBs without inherent ethical restrictions was also possible. Despite the expected low shear strength of Frasaco teeth based on a preliminary study, their resistance was sufficient to demonstrate differences in the mechanical behavior of the RBBs, as exclusive adhesive failure was verified only for RBBs manufactured with zirconia, a material with high toughness.

Transposing the findings to a clinical situation, it can be suggested that using an RBB made of ENA or SUP as their mode of failure led to the complete loss of the pontic, the removal of the retainer, and the manufacture of a new restoration would be necessary. In the case of Y-ZPT, loss of adhesion without RBB structural changes, a fact in line with the primary failure reported in the literature for this material, 33 37 would allow for an immediate new adhesive procedure. As for the ABS RBB, no information was found in the literature. This study suggests that its plastic deformation would allow the patient to have an appointment with the dentist before the pontic is lost, avoiding being toothless, an advantage over the other tested materials.

Concerning Medical ABS, its low melting point (105°C) makes it ideal for in-office equipment. It must be highlighted that if manufactured by extrusion-based fused deposition modeling (FDM), the orientation of the appendages influences its mechanical characteristics. Therefore, a careful design contemplating this aspect is necessary for an excellent final mechanical performance. 42 The significant benefits of FDM are low cost, rapid prototyping, and simplicity of procedure. 43 Complex geometries with a high concentration of stress should be avoided. However, if not possible, fabric from powder (SLS—selective laser sintering) should be preferred because the unused powder fills the gaps between the filaments. Still, this procedure makes the Medical ABS RBB much more expensive, increases postprocessing time, and requires extra equipment, such as powder removal stations. 26 Reducing the size of the extruder nozzle diameter and the thickness of the layers reduces the water absorption properties and increases the tensile and flexural strength of the specimens. 43

One cannot propose RBB as an option to rehabilitate the space of the MLIA without reflecting on occlusal function. Scientific literature focusing on occlusal efforts at the anterior level of the maxilla was not found, leading to a more embracing discussion. A study focusing on the maximum bite force (MBF) refers to a value of approximately 80 N (20% higher in bruxists) in individuals aged 22 to 48 years. 44 It varies with malocclusion, sex (higher in males), and age (increase until young adult age), decreasing significantly with vertical and transverse craniofacial and dental discrepancies, and with old age. 45 46 Patients with normal sagittal occlusion are expected to have more molar bite force than patients with malocclusions, with a magnitude two to three times greater in the molar region than in the anterior region. 47 A recent systematic analysis showed that the MBF ranged from 246.22 to 489.35 N and 5.69 to 16.1 kg in children and adolescents, respectively. 48 If a contact area of 1 mm ^2^ is assumed, respective values of 246 to 489 MPa and 0.56 to 158 MPa would be obtained. However, if the results from T-scan measurements of the occlusal contact area in MBF 49 are considered, revealing a mean value of 155 mm ^2^ for healthy young adults, the conversion would be to 0.3 to 3 MPa/mm ^2^ of contact area. Generally, a single-retainer RBB design should be preferred whenever canine guidance is present. However, with relatively short clinical crowns of the abutment teeth and limited bonding surface, a two-lingual retainer design might be preferred if a group function articulation is present. 8 25

Considering patients treated for MLIA by space opening reflection must be made because occlusal loads are higher than expected for the average patient whenever hypodivergence is present. 50 However, at the end of orthodontic treatment, an equilibrated occlusal function is mandatory, with a dispersed distribution of occlusal forces, thus theoretically reducing the adhesive stress on RBBs in the anterior maxilla.

Extrapolating the results of this study to clinical situations, Y-ZPT RBBs are the most suitable for MLIA rehabilitation, a finding consistent with the literature and that validates the choice of this material as the control material used in this study. However, more research is needed for newer zirconias with higher yttria contents because of their reduced toughness by almost half. Not testing them, instead of the tougher third-generation material, is a limitation of this study because newer compositions with higher yttria content, while improved aesthetically, have lower mechanical performance and are more susceptible to breakage. 15 16 Thickness, composition, microstructure, and cementing agent are crucial for the performance of the resistant tetragonal phase of monolithic zirconia, 51 advising caution when extrapolating results from research focusing on the longevity of older materials. 12 Although scarce, available randomized clinical trials (RCTs) using newer compositions have promising results. 38 Well-designed RCTs with large sample sizes are still needed to achieve more accurate results about the clinical success rate of different RBB designs in the anterior region, 24 25 knowing from the start that lower cantilever length and higher occlusocervical thickness significantly increases load to fracture values. 40 One well-controlled RCT, 38 some clinical studies, 8 52 53 and some systematic reviews 54 55 found in the literature revealed high survival rates and good clinical performance for the single-retainer zirconia RBBs from 18 months up to 15 years, with reported clinical results comparable or even better than those of the conventional fixed prosthesis and implant-supported crowns. 8 37 41 53

Still, patients with MLIA situations are frequently adolescents with ongoing maxillofacial growth, 2 and despite there is no sufficient evidence to either indicate or contradict the usage of dental implants in this age group, 7 its implantation requires positional modifications to attend periodontal and aesthetic factors, and they should be considered only under particular circumstances. 6 7 If infraocclusion of the replaced tooth occurs over time, new prosthetic restoration, new orthodontic treatment, or distraction osteogenesis may be necessary, 7 along with carefully planned rehabilitation treatment that should contemplate interim rehabilitation.

Whenever the option is short-term interim rehabilitation (orthodontic appliance removal or adaptation, periodontal remodeling or maturation, a short period between the end of orthodontic treatment and implant-supported crown placement, or even during the time of osseointegration of the implant), any other material is feasible and preferable because of more straightforward adhesive protocols, removal, or marginal adaptation for tissue management, if desired. For that purpose, printed ABS RBB is an interesting material. It can be fabricated quickly on the chairside at a low cost. 26 43 It requires only a hydrophobic resin for surface treatment, allowing an easy and quick cementation technique for minimally invasive rehabilitation. Further research using this or similar materials should be conducted in the future.

This study used a specific RBB design with a retainer on the palatal side of the central incisor. This design could raise constraints in cases of minimal interocclusal space due to sagittal or vertical discrepancies that may coexist in MLIA cases. Alternative approaches, such as employing a single retainer adhered to the buccal side of the central incisor or canine tooth, should be considered because of the thin dimensions of the retainer, which would not invade the buccal profile of the supporting tooth and a more straightforward cementation technique than the palatal one.

## Conclusion

RBBs made of Vita Enamic, Suprinity, Y-ZPT zirconia, or 3D-printed ABS can support physiological occlusal loads of the anterior maxilla. They can be used to rehabilitate MLIA in clinical situations. As long as the adhesive protocol is technically well executed, zirconia is the material of choice for definitive rehabilitation, as, due to its mechanical resistance, occasional adhesive failure without structural loss allows for immediate new cementation. If needed, ABS, ENA, and SUP are more suitable for interim RBBs because of the advantage of easier removal and in-mouth adaptation. The option for each material depends on the estimated time for use (temporary or permanent rehabilitation) and the necessity of removal for orthodontic or surgical techniques.
